# 391. Modified Human Immunodeficiency Virus Testing Algorithm in Rural Missouri (MOdified HIV testing algorithm)

**DOI:** 10.1093/ofid/ofae631.126

**Published:** 2025-01-29

**Authors:** Andres Bran Acevedo, Amy Bowers, Dima Dandachi, Christian Rojas-Moreno

**Affiliations:** University of Missouri, Columbia, Missouri; University of Missouri Healthcare, Columbia, MO; University of Missouri - Columbia, Columbia, Missouri; University of Missouri- Columbia, Columbia, Missouri

## Abstract

**Background:**

Universal screening for human immunodeficiency virus (HIV) is essential for timely and accurate diagnosis of HIV. The Centers for Disease Control and Prevention (CDC) recommends an algorithm that begins with HIV-1/HIV-2 Ag/Ab combination assay (HIV screening), which if reactive, is followed by HIV-1/HIV-2 antibody differentiation assay. If the differentiation assay is negative (discordant results), nucleic acid test for HIV RNA should follow. The University of Missouri Health Care (MUHC) sends the antibody differentiation assay to a reference laboratory, leading to delays in turn-around-time (TAT). As MUHC performs HIV-1 RNA testing in-house, we modified our own HIV screening algorithm to reflex HIV-1 RNA testing following a reactive HIV screening, with the goal of reducing TAT.

Human Immunodeficiency Virus Screening Algorithm

**Figure:**
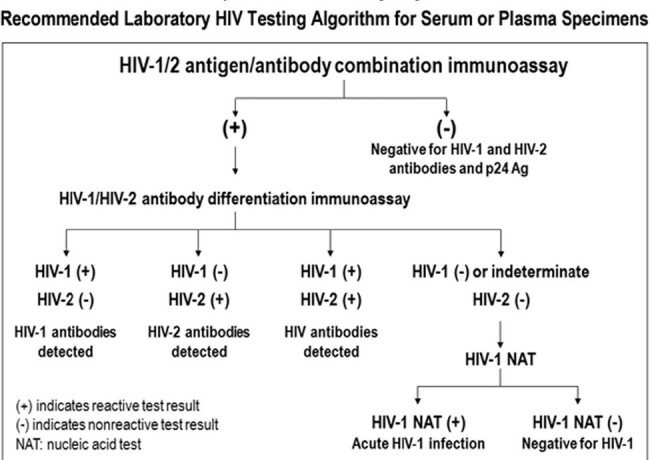

The above is the screening algorithm for human immunodeficiency virus (HIV) recommended by the Centers for Disease Control and Prevention utilized during period 1.

**Methods:**

Data from positive HIV screening tests was collected from two periods: period 1 (1/1/2021-12/29-2022), in which MUHC followed the screening algorithm recommended by the CDC (Figure 1); and period 2 (12/30/2022-12/31/2023) in which MUHC followed the Modified HIV testing algorithm (Figure 2). In both algorithms, if the test that follows a reactive HIV screening is positive, the patient is determined to have confirmed HIV infection. If negative, additional testing is performed to determine if reactive HIV screening was false positive or true positive. The TAT were collected and the differences in TAT between the two periods were analyzed using a two-sample t-test.

MOdified Human Immunodeficiency Virus Screening Algorithm

**Figure:**
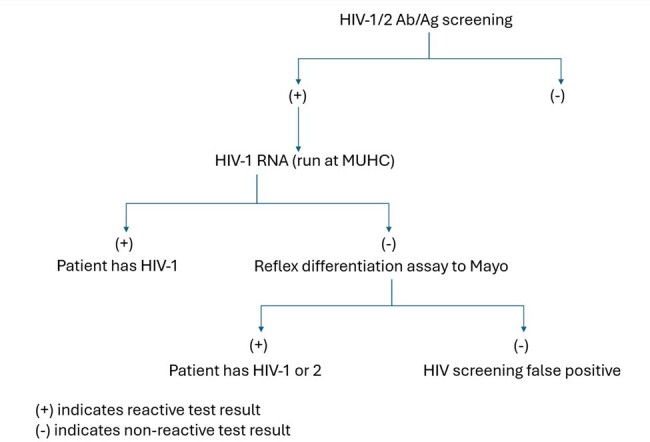

Above is the modified HIV screening algorithm utilized during period 2 in which the HIV-1 RNA is the first test following the reactive HIV screening test.

**Results:**

47 positive HIV screenings tests were analyzed in period 1 and 66 in period 2. The mean TAT between a reactive HIV screening and the differentiation assay result in period 1 was 3.6 days, whereas the TAT between a reactive HIV screening and the HIV RNA result in period 2 was 2.3 days (*p* 0.04). A higher percentage of tests were resulted in 1-3 days in period 2 compared to period 1 (77.2% vs 68.1%).

Turn Around Time Comparison

**Figure:**
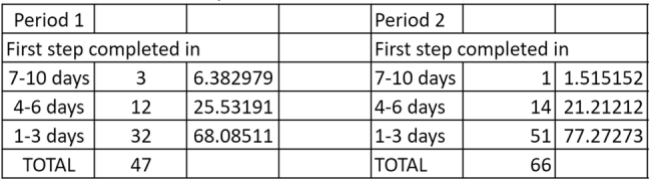

On the left is the data from first period where University of Missouri Healthcare utilized the CDC recommended algorithm. The data on the right is from the second period utilizing the modified algorithm.

**Conclusion:**

At our institution, after modification of the HIV testing algorithm to reflex RNA testing after a reactive HIV screening, patients received a faster confirmation of HIV diagnosis compared to CDC’s algorithm. This intervention could expedite linkage to care and initiation of antiretroviral therapy, especially at small and midsize institutions that often do not have the differentiation assay in-house.

**Disclosures:**

**Dima Dandachi, MD, MPH**, Gilead Sciences: Grant/Research Support|ViiV Healthcare: Grant/Research Support|ViiV Healthcare: Honoraria

